# Clusterin role in hepatocellular carcinoma patients treated with oxaliplatin

**DOI:** 10.1042/BSR20200071

**Published:** 2020-02-20

**Authors:** Xiumei Wang, Yongqiang Liu, Qiong Qin, Ti Zheng

**Affiliations:** 1 Department of Oncology, Inner Mongolia Autonomous Region Cancer Hospital and The Affiliated People’s Hospital of Inner Mongolia Medical University, Hohhot, Inner Mongolia Autonomous Region, 010059, China; 2 Department of General Surgery, Inner Mongolia Autonomous Region Cancer Hospital and The Affiliated People’s Hospital of Inner Mongolia Medical University, Hohhot, Inner Mongolia Autonomous Region, 010059, China; 3 Department of Oncology, People’s Hospital of Da La Te Banner, Inner Mongolia Autonomous Region, 014000, China

**Keywords:** Clusterin, Hepatocellular carcinoma, Oxaliplatin resistance

## Abstract

**Aim:** To explore the prognostic value of *clusterin* (*CLU*) in hepatocellular carcinoma (HCC) patients treated with oxaliplatin (OXA).

**Methods:** Relative expression of plasma *CLU* mRNA was examined via fluorescence quantitative real-time PCR (qRT-PCR), and CLU protein level in tissue samples was detected through immunohistochemistry. Chi-square test was used to analyze the relationship between *CLU* mRNA expression and clinical features of HCC patients treated with OXA. Kaplan–Meier method was performed to assess overall survival for the patients, and prognostic value of *CLU* in HCC patients was estimated via Cox regression analysis.

**Results:** *CLU* expression in plasma and tissue specimens was significantly higher among HCC patients than in non-malignant controls (*P* < 0.001 for both). Moreover, elevated *CLU* mRNA was closely related to tumor stage, lymph node metastasis and response to OXA (*P* < 0.05). HCC patients with high *CLU* expression showed poor response to OXA. In addition, low *CLU* levels predicted long overall survival time among the study subjects (20.8 vs. 36.6 months, *P* < 0.001). *CLU* was an independent prognostic indicator for HCC patients treated with OXA (HR = 2.587, 95%CI = 1.749–3.828, *P* < 0.001).

**Conclusion:** *CLU* may be a novel prognostic marker for HCC patients treated with OXA.

## Introduction

Hepatocellular carcinoma (HCC), accounting for 70–85% of all liver cancer cases, stands for a leading reason of tumor-relevant deaths around the world [1,2]. However, only less than 30% of HCC patients have the opportunity to receive curative treatments such as liver transplantation, surgical resection and ablation therapy, because most patients have entered into advanced stages at initial diagnosis [3]. Therefore, transcatheter hepatic arterial chemoembolization (TACE) and systemic chemotherapy represent main treatments for HCC [4]. Oxaliplatin (OXA), a third-generation platinum-derived chemotherapeutic agent, induces cell death through platinum-DNA adducts, and is more effective in inhibiting DNA replication than other platinum compounds [5,6]. OXA-based chemotherapy is widely used in treating several cancers, such as gastric cancer [7], colorectal cancer [8] and HCC [9]. However, clinical outcomes among HCC cases treated with OXA are varied, due to drug resistance. Therefore, finding key factors denoting HCC cases’ sensitivity to OXA will improve therapeutic effect of chemotherapeutic agents.

Secretory clusterin (s*CLU*), a heterodimeric secreted glycoprotein, is highly conserved, and its expression is observed in a variety of tissues and human fluids [10]. *Clusterin* (*CLU*) plays important roles in tissue remodeling, reproduction, lipid transport, complement regulation and apoptosis [11]. Growing evidences have demonstrated that the over-expression of *CLU* in malignancies may contribute to tumor progression [12,13]. Additionally, a report indicated that *CLU* enhances treatment resistance due to its antiapoptotic action [14]. Xiu and colleagues suggested that *sCLU* could strengthen OXA resistance through activating Akt pathway in HCC patients [15]. But the clinical effect of *CLU* in HCC is rarely reported.

Therefore, our study evaluated the prognostic value of *CLU* in HCC patients treated with OXA. We measured *CLU* expression for HCC patients before OXA treatment, and its connections with clinical features and response to OXA were also analyzed. Besides, we also estimated overall survival for the cases with varied *CLU* degrees, and Cox regression analysis was performed to estimate prognostic value of *CLU*.

## Materials and methods

### Patients and samples

About 104 cases were pathologically and clinically diagnosed with HCC in Inner Mongolia Autonomous Region Cancer Hospital and The Affiliated People’s Hospital of Inner Mongolia Medical University between October, 2018 and July, 2019, based on histopathological examination. Meanwhile, 60 healthy volunteers were selected as the control group. Fresh blood specimen from each participant was collected into EDTA tubes at 4°C, and then centrifuged for 5 min at 2500 rpm. Plasma specimens were kept at −80°C for later. No patients had received any chemotherapy or radiotherapy before the blood extraction. Besides, both HCC and adjacent normal tissue specimens were collected from HCC patients, and frozen in liquid nitrogen. Then, the tissue specimens were stored at −80°C for subsequent application.

The HCC patients were followed up for 5 years, every 3 months for the first year, every 6 months for the subsequent 2 years, and then annually. Clinical parameters including gender, age, tumor size or tumor stage, lymph node metastasis, serum AFP, vascular invasion, cirrhosis and recurrence were recorded when the patients were enrolled in the study, via questionnaire and their medical records. The present study was approved by the ethics committee of the Inner Mongolia Autonomous Region Cancer Hospital and The Affiliated People’s Hospital of Inner Mongolia Medical University, and written informed consents were also signed by all patients or their families.

### RNA extraction and qRT-PCR

Total RNA was extracted from plasma samples using Trizon reagent (Invitrogen, Carlsbad, CA) according to the manufacturer’s instructions. The concentration of total RNA was examined through UV absorbance (A260/A280), and 1% agarose gel electrophoresis was employed to test the quality of RNA sample. Total RNA samples with high quality, referring TO those with a concentration of 200 ng/μl, were used for synthesizing the first chain of cDNA, which was performed using Prime Scrip RT reagent kit (Takara, China). Fluorescence quantitative real-time polymorphism chain reaction (qRT-PCR) was performed via SYBR Green assay (Takara, China). β-Actin was an internal control. Data were analyzed through 2^−∆∆Ct^ method. Primer sequences were displayed in Table 1.

**Table 1:** The sequences of the primers used in the present study

Name		Sequences
*CLU*	Forward	ATTCATACGAGAAGGCGACG
	Reverse	CAGCGACCTGGAGGGATT
*β-Actin*	Forward	GAAATCGTGCGTGACATTAA
	Reverse	AAGGAAGGCTGGAAGAGTG

### Immunohistochemistry (IHC)

Both HCC and adjacent normal tissue specimens were cut into sections reaching a thickness of 4μm, and then deparaffinized in 65°C oven. The slides were incubated for 2 min in citrate buffer (10 mM) at 100°C for antigen retrieval and then blocked by goat serum. Next, the sections were incubated with anti-clusterin antibody (1:300, Sino Biological Inc., Catalog No. 11297-R210) or anti-GAPDH antibody (1:1000,Sino Biological Inc., Catalog No. 100242-T08) at 4°C overnight. Then, the sections were incubated with horseradish peroxidase (HRP) conjugated anti mouse secondary antibody (1:200, Abcam, U.S.A.) at room temperature for 30 min. Finally, the sections were stained using 3,3’-diaminobenzidine (DAB) solution, followed by counterstaining with hematoxylin.

Staining results were analyzed by two physicians who were blind to the information about the slides. Staining range was scored according to the following standards: 0, (<5%); 1, (5–25%; 2, (26–50% positive tumor cells); 3, (51–75%); and 4, (>75%). Staining intensity was graded according to the following criteria: 0, nonstaining; 1, light yellow; 2, brownish yellow; and 3, brown. Final scores referred to the sum of staining range and staining intensity scores. Final score ≤3 stood for low expression and >3 meant high expression.

### OXA treatments

All patients received OXA treatment with a dosage of 100 mg/m^2^ on day 1 and 15 through a 2-h intravenous infusion and were pretreated with antiemetics. Treatment was repeated every 28 days [9]. Tumor response to OXA was evaluated by a radiation oncologist on the basis of response evaluation criteria in solid tumors (RECIST) version 2.

### Statistical analysis

Continuous data were presented as mean ± standard deviation (SD), and their comparison between two groups was carried out using Student’s *t* test. Correlation of *CLU* mRNA level with clinical characteristics of HCC patients was evaluated through Chi-square test. Overall survival of the patients was analyzed via Kaplan–Meier method with log rank test. Cox regression analysis was performed to estimate prognostic significance of *CLU* in HCC patients treated with OXA. All statistical analyses were carried out in SPSS 18.0 software. *P* < 0.05 stood for the presence of statistical significance of results.

## Results

### Clinical information of HCC patients

About 104 HCC patients including 58 men and 46 women were recruited in our research. The average age of included patients was 56.9 years old. Of the patients, 46 exhibited complete response (CR) or partial response (PR), showing an overall response rate of 44.2%. Table 2 described clinical profiles for all enrolled subjects.

**Table 2:** The association between *CLU* expression and clinical features in HCC

Characteristics	Total number (*n*)	*CLU* expression		χ^2^	*P*
		High (*n*)	Low (*n*)		
Gender				0.122	0.727
Male	58	27	31		
Female	46	23	23		
Age				0.732	0.392
≥55	60	31	29		
<55	44	19	25		
Tumor size				0.555	0.456
≥5	46	24	22		
<5	58	26	32		
Tumor stage				7.550	0.006
I+II	52	18	34		
III+IV	52	32	20		
Lymph node Metastasis				9.861	0.002
Yes	52	33	19		
No	52	17	35		
Serum AFP				0.594	0.441
≥200 ng/dl	50	26	24		
<200 ng/dl	54	24	30		
Vascular invasion				0.472	0.492
Absent	72	33	39		
Present	32	17	15		
Cirrhosis				2.143	0.143
Yes	61	33	28		
No	43	17	26		
Recurrence				1.135	0.287
Yes	43	18	25		
No	61	32	29		

### Expression of CLU and its association with clinical features of HCC patients

**Figure 1:**
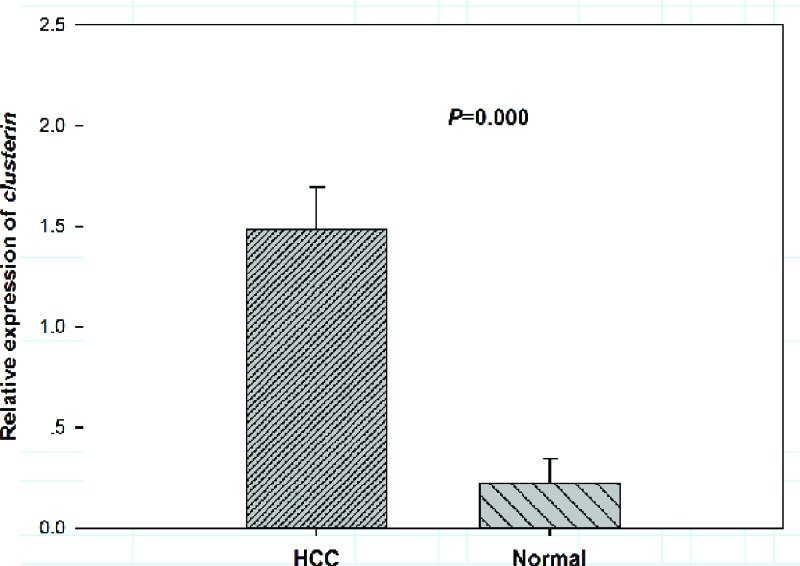
Relative expression of plasma *CLU* in HCC cases and normal controls (β-actin as normalized control)

Relative expression of *CLU* mRNA in patients with HCC was determined via qRT-PCR. Accordingly, plasma *CLU* expression was higher in HCC cases than in normal controls (1.48 ± 0.22 vs. 0.22 ± 0.12, *P* < 0.001, Figure 1).

**Figure 2:**
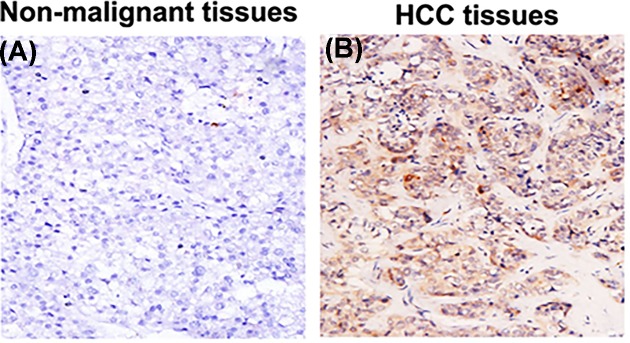
Representative IHC images for negative expression of CLU protein in non-malignant tissues (**A**) and for positive expression of CLU protein in HCC tissues (**B**)

In addition, the expression of CLU protein in HCC tissues and non-malignant tissues was also estimated using IHC method. The results suggested that the expression of CLU protein in HCC tissues was significantly higher and the percentage of positively stained cells was as high as 94.2% (98/104); while CLU protein expression in non-malignant tissues was relatively weaker and the proportion of positively stained cells was only 14.4% (15/104). The difference between two sides was significant (*P* < 0.001, Figure 2).

The patients were classified into two groups on the basis of their average *CLU* mRNA levels. Chi-square test indicated that *CLU* level was closely related to tumor stage (*P* = 0.006) and lymph node metastasis (*P* = 0.002), but not to gender, age, tumor size, serum AFP, vascular invasion, cirrhosis or recurrence (*P* > 0.05 for all). All data were listed in Table 2.

### Response rate and CLU expression in HCC patients treated with OXA

Patients with low *CLU* mRNA expression emerged more frequently in CR+PR group than in SD+PD group. It suggested that *CLU* mRNA levels were obviously related to response to OXA treatment in HCC patients (Table 3, *P* = 0.001). Subjects possessing high expression exhibited high OXA resistance, while low ones showed well response rate.

**Table 3:** Response rate of HCC patients to OXA treatment according to *CLU* expression

Response rate	CR+PR	SD+PD	χ^2^	*P*
*CLU* high expression (*n*)	14 (13.5%)	36 (34.6%)		
*CLU* low expression (*n*)	32 (30.8%)	22 (21.2%)	10.284	0.001
Total number (*n*)	46 (44.2%)	58 (55.8%)		

Abbreviations: CR, complete response; PD, progressive disease; PR, partial response; SD, stable disease.

### Overall survival analysis for HCC patients treated with OXA

**Figure 3:**
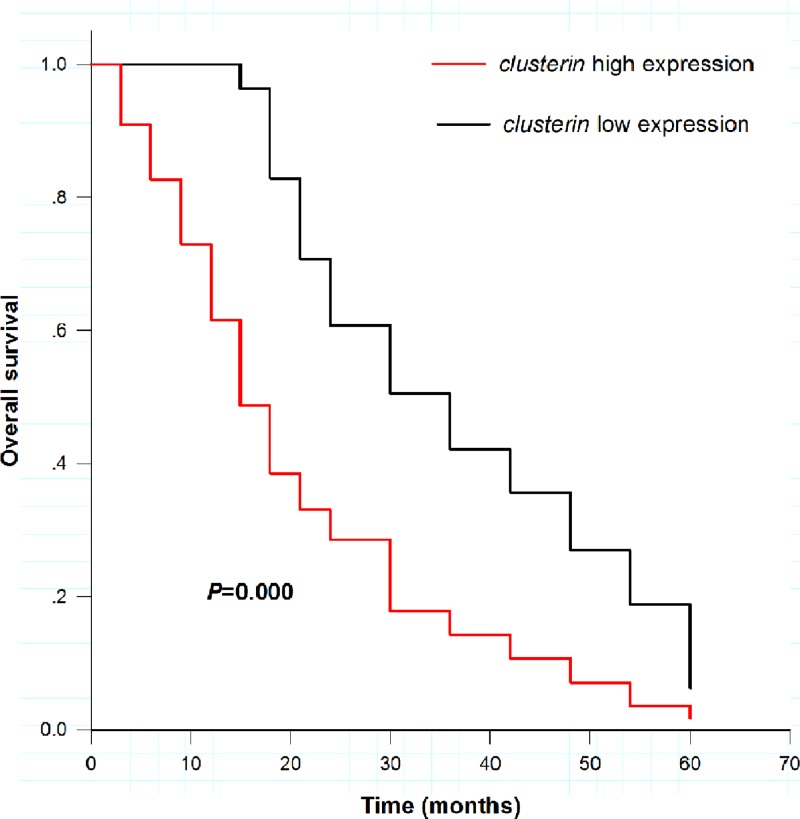
Overall survival of HCC cases treated with OXA stratified by *CLU* expression Cases showing high expression suffered poor survival rate (red line), while those in low expression group had well survival rate (black line), showing significant difference (Log rank test, *P* < 0.001)

The average overall survival time was 20.8 months in HCC patients with high *CLU* expression, and 36.6 months in patients with low expression (Figure 3), showing remarkable difference (Log rank test, *P* < 0.001). Cox regression analysis results suggested that *CLU* was related to the outcomes of HCC patients treated with OXA (*P* < 0.001). *CLU* could be an independent prognostic biomarker (Table 4, HR = 2.587, 95%CI = 1.749–3.828, *P* < 0.001).

**Table 4:** Univariate and multivariate analysis for prognostic factors in HCC patients

Characteristics	Univariate analysis			Multivariate analysis		
	HR	95%CI	*P*	HR	95%CI	*P*
*CLU* (high vs. low)	2.575	1.755–3.778	0.000	2.587	1.749–3.828	0.000
Tumor size (>5 cm vs. <5 cm)	1.107	0.764–1.604	0.593	1.059	0.718–1.561	0.773
Tumor stage (III+IV vs. I+II)	1.122	0.777–1.619	0.540	0.986	0.667–1.459	0.944
Lymph metastasis (yes vs. no)	1.021	0.708–1.473	0.911	1.074	0.724–1.594	0.721

## Discussion

Abnormal expression of *CLU* has been reported to be associated with treatment resistances in several malignancies. For example, Watari and colleagues have found significant relationship between *CLU* over-expression and poor overall survival in advanced cervical cancer patients receiving curative intended radiotherapy [16]. Brent et al. have indicated that decreased serum *CLU* level predicted longer survival time in castration-resistant prostate cancer cases [17]. In the study of He et al., *CLU* acted as a promising biomarker for esophageal squamous cell carcinoma patients who were resistant to chemoradiotherapy [18]. In the present study, the influence of *CLU* on OXA resistance in HCC patients was evaluated.

Expression patterns of *CLU* in HCC patients before OXA treatment were detected in the present study. The results demonstrated that *CLU* mRNA and protein levels significantly increased in HCC tissues compared with non-malignant tissues. Moreover, high serum *CLU* levels were related to advanced tumor stage and positive lymph node metastasis. Moreover, HCC patients with high expression of *sCLU* showed poor response to OXA treatment. Previous study has reported that *sCLU* could enhance OXA resistance via activating phosphorylated Akt in HCC [15]. *CLU* was associated with OXA resistance in HCC patients.

*CLU* was associated not only with OXA resistance, but also with resistances to other chemotherapeutic agents. Hassan et al. have indicated that *CLU* was associated with paclitaxel resistance in ovarian cancer patients [19]. While in melanoma cells, *CLU* could regulate their resistance to cytotoxic drugs *in vitro* and *in vivo*. Moreover, knocking-down *CLU* might provide a novel way to overcome drug resistance in melanoma [20]. In human osteosarcoma cells, *sCLU* down-regulation could enhance individual sensitivity to cisplatin via activating ERK1/2 signals [21]. Besides, *CLU* expression was also related to irradiation and oxidative stress [22]. All of these studies indicated that *CLU* was a broad-resistant gene and might be a potential therapy target in cancer management.

Prognostic significance of *CLU* in malignancies has been reported in previous studies. And *sCLU* over-expression was detected in colon cancer cells, and further research indicated that *sCLU* was a potential diagnostic marker for colorectal cancer [23]. Lokamani and colleagues indicated that *CLU* might serve as a potential marker to distinguish cervical neoplasis with borderline morphology features [24]. Abnormal expression of *CLU* has also been reported in patients with transitional bladder cell carcinoma, which might act as a diagnostic and prognostic biomarker for the disease [25]. In the present research, we explored prognostic significance of *CLU* in HCC patients treated with OXA. The results demonstrated that high expression of *CLU* meant poor overall survival among HCC cases. *CLU* could predict HCC patients’ prognosis who were treated with OXA.

Several limitations in our study should be stated here. First, the sample size was relatively small that might lead to low statistical power and affect the accuracy of our results. Second, all patients were collected from one hospital, which might cause bias into final results. Third, molecular mechanisms of *CLU* affecting resistance to OXA among HCC patients remained unclear. Fourth, tumor stage and lymph node metastasis were closely associated with CLU expression, and subgroup analysis based on these factors will increase the stringency of data synthesis. However, sample size was not large enough to perform such subgroup analysis. In addition, how CLU changed over time and after each treatment should be explored as well, which might help us understand the mechanism of CLU affecting OXA treatment in HCC patients. In further investigations, *in vitro* and *in vivo* experiments should be carried out to address the issue.

## Conclusion

In conclusion, *CLU* over-expression is observed in plasma specimens from HCC cases, and its levels are related to tumor stage and lymph node metastasis. Besides, *CLU* is related to OXA resistance in HCC patients, and its high expression associates with low response rate. *CLU* could predict overall survival for HCC patients treated with OXA, which may offer a new way to overcome therapeutic resistance in the cancer management.

## Abbreviations

*CLU*: *clusterin*
CR: complete response
DAB: 3,3’-diaminobenzidine
HCC: hepatocellular carcinoma
HRP: horseradish peroxidase
IHC: immunohistochemistry
OXA: oxaliplatin
PR: partial response
qRT-PCR: quantitative real-time PCR
